# Effect of the Deletion of Genes Encoding Proteins of the Extracellular Virion Form of Vaccinia Virus on Vaccine Immunogenicity and Protective Effectiveness in the Mouse Model

**DOI:** 10.1371/journal.pone.0067984

**Published:** 2013-06-13

**Authors:** Clement A. Meseda, Joseph Campbell, Arunima Kumar, Alonzo D. Garcia, Michael Merchlinsky, Jerry P. Weir

**Affiliations:** Division of Viral Products, Center for Biologics Evaluation and Research, United States Food and Drug Administration, Rockville, Maryland, United States.; Univ. of Texas HSC at San Antonio, United States of America

## Abstract

Antibodies to both infectious forms of vaccinia virus, the mature virion (MV) and the enveloped virion (EV), as well as cell-mediated immune response appear to be important for protection against smallpox. EV virus particles, although more labile and less numerous than MV, are important for dissemination and spread of virus in infected hosts and thus important in virus pathogenesis. The importance of the EV A33 and B5 proteins for vaccine induced immunity and protection in a murine intranasal challenge model was evaluated by deletion of both the *A33R* and *B5R* genes in a vaccine-derived strain of vaccinia virus. Deletion of either *A33R* or *B5R* resulted in viruses with a small plaque phenotype and reduced virus yields, as reported previously, whereas deletion of both EV protein-encoding genes resulted in a virus that formed small infection foci that were detectable and quantifiable only by immunostaining and an even more dramatic decrease in total virus yield in cell culture. Deletion of *B5R*, either as a single gene knockout or in the double EV gene knockout virus, resulted in a loss of EV neutralizing activity, but all EV gene knockout viruses still induced a robust neutralizing activity against the vaccinia MV form of the virus. The effect of elimination of A33 and/or B5 on the protection afforded by vaccination was evaluated by intranasal challenge with a lethal dose of either vaccinia virus WR or IHD-J, a strain of vaccinia virus that produces relatively higher amounts of EV virus. The results from multiple experiments, using a range of vaccination doses and virus challenge doses, and using mortality, morbidity, and virus dissemination as endpoints, indicate that the absence of A33 and B5 have little effect on the ability of a vaccinia vaccine virus to provide protection against a lethal intranasal challenge in a mouse model.

## Introduction

Smallpox was officially declared eradicated by the World Health Organization in 1980, and routine vaccination against smallpox no longer recommended except for select groups (e.g., laboratory workers working with poxviruses) [1]. While variola virus, the causative agent of smallpox, is officially retained at two World Health Organization (WHO) collaborative centers: The Centers for Disease Control and Prevention, Atlanta, Georgia, United States, and The State Research Center of Virology and Biotechnology, Novosibirsk, Russia, it is not known with certainty whether there exist other undeclared sources of the virus [2]. Thus, there remains some concern of a possible re-emergence of smallpox due to an inadvertent release of variola from the laboratory or from deliberate use of the virus as a bioweapon. In addition, a number of orthopoxviruses that infect different animal species are known to be potential sources of zoonotic infections in humans, including most notably monkeypox virus [3-5], but also other poxviruses such buffalopox [6] and cowpox [7,8]. Consequently, the availability of safe and effective smallpox vaccines remains a high public heath priority, as a significant portion of the population is susceptible to orthopoxvirus infection resulting from cessation of routine smallpox vaccination.

Despite their efficacy as prophylactic vaccines against smallpox, traditional smallpox vaccines, which are strains of live, replicating vaccinia virus, have been associated with some rare but serious adverse reactions in some vaccinees [9]. Somewhat more recently, myocarditis and pericarditis have also been noted following smallpox vaccination [10,11]. Because of such complications, efforts have been made to develop safer, less reactogenic new-generation smallpox vaccines.

The safety profile of candidate new smallpox vaccines can be established in the clinic, but in the absence of clinical smallpox, evaluation of efficacy of new-generation smallpox vaccines poses a challenge. Efficacy evaluation will rely heavily on data obtained from appropriate animal models, as well as bridging of preclinical immunogenicity and efficacy data to immunogenicity data obtained in clinical studies [12]. Complicating efficacy evaluation, particularly for smallpox vaccines that are fundamentally different from vaccines used for eradication of smallpox, is that the correlates of protective immunity to smallpox are not known [13]. Nevertheless, numerous studies have implicated the importance of an antibody response elicited by vaccination for protection against smallpox. There are two antigenically distinct infectious forms of vaccinia virus, the mature virion (MV) and the enveloped virion (EV) (also known as the intracellular mature virion [IMV] and extracellular enveloped virion [EEV], respectively) [14]. Antibodies to both forms of the virus appear to contribute to protection. EV virus particles, although more labile and less numerous than MV, are important for dissemination and spread of virus in infected hosts and thus important in virus pathogenesis [15].

The *A33R* and *B5R* genes of vaccinia virus encode the EV A33 and B5 proteins, respectively. Antibodies to each protein inhibit virus spread in cell culture and B5 antibody neutralizes EV infectivity. Further, immune responses to A33 [16,17] and B5 [16] also elicit full or partial protection in various animal models and the majority of the EV-neutralizing activity in human vaccinia immunoglobulin (VIG) is directed at B5 [18]. In addition, in animal models in which a combination of both MV and EV antigens are used for immunization, a more robust protection is achieved than if antigens from only one of the forms of virus are used for immunization [19,20]. Taken together, the data suggest that A33 and B5 may be important vaccine components of an effective vaccine. However, it is not clear if the antibody response to the A33 and B5 is absolutely required for the protection afforded by vaccination with smallpox vaccines. The aim of the present work was to determine the effect of the deletion of both the *A33R* and *B5R* genes on vaccine induced immunity and protection, using a virulent vaccinia virus challenge in a mouse model.

## Materials and Methods

### Ethics Statement

Male BALB/cByJ mice (4–5 weeks old) were obtained from the Jackson Laboratory, Bar Arbor, Maine. Mice were housed at an animal facility provided by the Center for Biologics Evaluation and Research (CBER). Care and handling of animals were performed according to guidelines provided by the Animal Research Advisory Committee, National Institutes of Health. Mice were fed with sterile feed and drinking water, and were routinely cared for by the Division of Veterinary Services, CBER. The animal study protocol was approved by the CBER Animal Use and Care Committee.

### Cells and Viruses

BSC-1 cells (ATCC CCL-26), RK-13 cells (ATCC CCL-37), and BSC-40 cells (ATCC CRL-2761) (a derivative of BSC-1) were grown and maintained in Dulbecco’s modified Eagles’s medium (DMEM) containing 10% fetal bovine serum (FBS), and 50 µg/ml gentamicin. BSC-40 cells were obtained from Dr. Bernard Moss, National Institutes of Health (NIH), and were routinely used to determine vaccinia virus titer.

A clonal isolate of vaccinia virus, DV-3, was isolated by plaque purification from the Dryvax virus seed stock described previously [21]. DV-3 was prepared from infected BSC-1 cells and virus titer was determined using BSC-40 cells. Vaccinia virus strains WR and IHD-J, as well as recombinants WR-luc and IHDJ-luc, were prepared from infected BSC-40 cells as previously described [22,23]

### Plaque Assay and Immunostaining

Confluent monolayers of BSC-40 cells in 6-well tissue culture plates were infected with diluted virus suspensions. Control wells were mock-infected with DMEM medium. After 2 hours of incubation at 37 ^°^C, an overlay of 2 ml growth medium containing 0.5% carboxymethyl cellulose (CMC) was added to each well, and plates were re-incubated for 2 to 7 days (as necessary, depending on the virus). For crystal violet staining, the CMC overlay was aspirated and a solution of 0.5% crystal violet containing 25% formalin (fixative) was added to each well. After 30 minutes of staining, plates were rinsed with water to reveal plaques.

For detection of plaques by immunostaining, cells were rinsed with PBS after the removal of the CMC overlay, and fixed with a cold solution of acetone/methanol (1:1) for 10 minutes. A blocking solution (3% FBS in PBS) was added to wells, and rocked for 1 hour at room temperature. The primary antibody, a rabbit anti-vaccinia antibody (YVS8101; Accurate Chemicals, Westbury, New York) diluted to 1:500 in blocking solution was added, and plates were rocked for additional 1 hour at room temperature. Cells were washed 3 times with PBS, and a secondary antibody, an Alkaline phosphatase-conjugated goat anti-rabbit antibody was added at 1:7,500 dilution. After 1 hour incubation, the plates were washed 3 times with PBS, and the Western blue stabilized substrate for alkaline phosphatase (Promega Corp., Madison, Wisconsin) was added. After 5–10 minutes of rocking at room temperature, excess substrate was rinsed off with water. The number of plaques or immunostained foci were counted and virus titer was calculated. Images of crystal violet-stained and immunostained plaque were scanned with HP Scanjet 5590 scanner (Hewlett-Packard, Palo Alto, CA).

### Construction of recombinant knockout viruses B5Rko, A33Rko, and A33R/B5Rko

Recombinant vaccinia viruses deleted of the *B5R* (B5Rko), *A33R* (A33Rko), or both *A33R* and *B5R* (A33R/B5Rko) were constructed by homologous recombination, using a gene knockout strategy as previously described [24]. A 788bp fragment containing the sequence of the enhanced green fluorescent protein (GFP) under the vaccinia virus P11 promoter was amplified from plasmid pLW44 (a gift from Linda Wyatt from the Bernard Moss Laboratory, NIH) with a pair of primers corresponding to coordinates 924–974 and the reverse complement of coordinates 1661–1710 of pLW44 [25]. Primers for the generation of flanking sequences of *A33R* or *B5R* by PCR were designed based on the published sequence of vaccinia virus strain WR (GenBank accession number NC_006998), and DNA isolated from a plaque-purified clonal isolate of Dryvax, DV-3, was used as the template.

In constructing B5Rko, the left flanking sequence was generated with coordinates 167859–167886 and the reverse complement of coordinates 168342–168373 as primers, with the latter containing the reverse complement of coordinates 924–944 of pLW44. The right flanking sequence was generated with coordinates 169334–169363 containing coordinates 1690–1710 of pLW44 and the reverse complement of coordinates 169843–169869 as primers. The left and right fragments were combined with the GFP fragment amplified from pLW44, and using the outermost primers of the *B5R* flanking sequences to amplify an approximately 1.8 kb DNA fragment by PCR. This fragment was cloned into the plasmid pCR2.1 (Invitrogen, Carlsbad, CA) to generate pTRIKOB5. The plasmid was transfected into BSC-1 cells infected with DV-3 using FuGENE (Promega, Madison, WI) and virus plaques expressing GFP were isolated, and plaque-purified.

Recombinant A33Rko was constructed by a similar method as above. The left flanking sequence of *A33R* was amplified with coordinates 142420–142444 and the reverse complement of coordinates 143299–143328 as primers, with the latter containing the reverse complement of coordinates 924–944 of pLW44. The right flank was generated using coordinates 145886 - 143910 containing coordinates 1690–1710 of pLW44 on the 5 end and the reverse complement of coordinates 144473–144505, as primers. The left and right fragments were combined with the GFP fragment amplified from pLW44, and using the outermost primers of the *A33R* flanking sequences to amplify a 2.6kb DNA fragment by PCR. This was cloned into the plasmid pCR2.1 to generate pTRIKOA33. Recombinant virus plaque expressing GFP (A33Rko) was generated as described above, and plaque-purified.

The double recombinant virus vA33RB5Rko was constructed by modification of B5Rko. A 690 base pair coding sequences for the DsRED monomer was amplified from pDsRED Monomer (pT3794-5) (Clontech Laboratories, Inc., Mountain View, CA) by PCR, using nucleotides 289 to 309 of pT3794 (Clonetch Laboratories) containing a *Nde*I site and the reverse complement of nucleotides 952 to 979 of pT3794 containing a *Not*I site. The DsRED PCR fragment was cloned into pCR2.1 to obtain pREDleft, a plasmid where the side of the DNA fragment containing the *Nde*I restriction site was closest to the *Xba*I restriction site in pCR2.1. A pair of complementary sequences of nucleotide sequences corresponding to the vaccinia virus promoter in pLW44 (nucleotides 942 to 967), and containing *Nde*I and *Xba*I overhangs and an *Asc*I site were annealed and ligated with pREDleft that has been linearized with *Nde*I and *Xba*I to produce pVVDsRED. This plasmid contains the dsRED monomer under the control of the vaccinia virus promoter used in pLW44. The left flanking sequence of *A33R* was amplified using the reverse complement of coordinates 144473–144505 containing an *Xba*I site, and coordinates 143886–143910 containing an *Asc*I site. The PCR product was cloned into pCR2.1 to generate pTAA33Rleft, and sub-cloned as a 620 bp *Asc*I to *Xba*I fragment into pVVDsRED to generate PVVDsREDA33left. The right flanking sequence of *A33R* was amplified with a pair of primers corresponding to coordinates 142420–142444 and the reverse complement of coordinates 143299–143328, containing a *Hin*dIII and a *Kpn*I site, respectively. The PCR product was cloned into pCR2.1 to generate pTAA33Rright. A 910 bp fragment containing the right flanking sequence of *A33R* was excised from pTAA33Rleft as a *Hin*dIII and *Kpn*I fragment and inserted into PVVDsREDA33left to produce PTRIA33DsRED. The plasmid PTRIA33DsRED was transfected into BSC-1 cells infected with B5Rko using FuGENE, and recombinant virus plaques expressing both DsRED and GFP (A33R/B5Rko) were isolated and plaque purified.

The structure of the three recombinant viruses was confirmed by PCR assay using oligonucleotide primers that produced DNA fragments that could distinguish among wild type DV-3, A33Rko, B5Rko, and A33RB5Rko. Titers for A33Rko and B5Rko recombinants were determined by plaque assay on BSC-1 cells using crystal violet staining after 48 hours. The vA33RB5Rko double knockout was titered by plaque assay and plaques were detected by immunostaining.

### Construction of Recombinant WR-luc and IHDJ-luc Challenge Viruses

Recombinant vaccinia viruses strain WR and strain IHD-J expressing the firefly luciferase (WR-luc and IHDJ-luc, respectively) were constructed by insertion of the luciferase gene at the locus of the equivalent of the *CP77* gene of cowpox virus. The *CP77* gene is a host range gene that is fragmented or absent in vaccinia virus strains [26,27]. The luciferase gene from pEL/tk-luciferase, a plasmid containing the firefly luciferase gene under the control of the vaccinia synthetic early/late promoter [28], was amplified by PCR and cloned into the pCR2.1 vector (Invitrogen) to generate pCRluciferase. The luciferase gene under the vaccinia virus early/late promoter was amplified by PCR from pCRluciferase using a pair of primers corresponding to nucleotides 235–285 of pCR2.1 and the reverse complement of nucleotides 291–341. Primers for the generation of flanking sequences of *CP77* gene locus by PCR were designed based on the published sequence of vaccinia virus strain WR (GenBank accession number NC_006998), and DNA isolated from a VV–WR was used as the template. The left flanking sequence of the fragmented *CP77* gene equivalent in VV–WR was generated by PCR using coordinates 11527–11562 and the reverse complement of coordinates 12547–12573 as primers, with the latter containing nucleotides 235–262 of pCR2.1. Similarly, the right flanking sequence of the *CP77* locus was generated using the reverse complement of coordinates13483 -13515 and coordinates12673 -12607 as primers, with the latter containing nucleotides 313 to 341 of pCR2.1. The products from the three PCR were purified and combined as template for the amplification of a fragment that contains sequences of the left flank, luciferase, and the right flank, using a pair of primers from the outermost sequences of the right and left flanking sequences. This fragment contains one contiguous DNA fragment containing regions corresponding to the vaccinia *CP77* host range gene interrupted by the luciferase gene under control of the vaccinia early/late promoter. This fragment was recombined with IHD-J-GFP, a vaccinia virus IHD-J strain containing the GFP gene inserted into the site corresponding to the *CP77* gene to obtain IHDJ-luc. Similarly, the fragment was recombined with WR-GFP, a vaccinia virus WR strain containing the GFP gene inserted into the site corresponding to the *CP77* gene to obtain WR-luc. Virus clones not expressing GFP were isolated, purified, and screened for luciferase expression. IHDJ-luc was further verified by determining the nucleotide sequence of the viral *A34R* gene to confirm the presence of Glu151 and by observing plaque phenotype under liquid media after infection of BSC-1 cells in monolayers. The virulence of WR-luc and IHDJ-luc was shown to be similar to wild type WR and wild type IHD-J, respectively, by determining their 50% lethal dose (LD_50_) in mice, using the Reed and Muench method [29].

### Mouse Immunization and Virus Challenge

Vaccination of mice with vaccinia virus DV-3, recombinant viruses A33Rko, B5Rko, and A33/B5Rko, was performed by tail scarification as previously described [30]. Briefly, mice were anesthetized by intra-peritoneal injection of 20 µl/g body weight of a solution of 1x Avertin (2,2,2, -tribromoethanol dissolved in tert-amyl alcohol), and pricked/scratched at the base of the tail with a 25-gauge needle. Virus suspension containing the desired dose of virus (10^3^, 10^4^, or 10^5^ pfu) in 2 µl volume was applied to the inoculation site.

Vaccinia virus challenge of mice with the Western Reserve (WR) strain and the International Health Department J (IHD-J) strain, or recombinant WR-luc and IHDJ-luc, was performed by intranasal inoculation as previously described [31]. Mice were weighed and anesthetized with a solution of 1x Avertin. The appropriate challenge dose of 25- or 100-times the 50% lethal dose (25 or 100 LD_50_) of the challenge virus was suspended in endotoxin-free PBS, and 10 µl was applied into each nostril (the LD_50_s of the challenge viruses were 0.4 x 10^4^ pfu, 4.2 x 10^4^ pfu, 4.2 x 10^4^ pfu, and 3.2 x 10^4^ pfu for WR, WR-luc, IHD-J, and IHDJ-luc, respectively). Mice were observed and weighed daily for 10 to 12 days, and those that lost 25% of their original body weight were euthanized in accordance with the animal study protocol. In experiments where mice were challenged with recombinant WR-luc or IHDJ-luc, mice were weighed and in vivo imaging detection of luciferase expression was performed using the IVIS-50 system (Caliper Life Sciences [Xenogen], Alameda, CA) as previously described [32]. Images were captured under a charge-coupled device camera, and analyzed using the Living Image 3.2 software (Caliper Life Sciences, Hopkinton, MA) to quantify photon fluxes. The photon flux data were exported to Microsoft Excel for the computation of mean photon flux ± STDEV at each time point.

#### ELISA Assays

The detection of vaccinia-specific immunoglobulin G (IgG) against the MV form of vaccinia virus by enzyme-linked immunosorbent assay (ELISA) was performed using inactivated Dryvax as coating antigen for antibody capture. ELISA detection of anti-vaccinia EV A33 and B5 IgG using affinity-purified recombinant vaccinia A33 and B5 proteins [33] as coating antigens, respectively, was as previously described [23,30]. The endpoint titer was defined as the highest dilution of test serum that had an absorbance at 405 nm greater than that of the matched dilution of normal pre-bleed mouse serum and a value that was also ≥ 0.050.

### Vaccinia Virus PRNT

Serum samples obtained from mice were pooled by treatment group in each experiment and tested for virus neutralization by traditional plaque reduction neutralization test (PRNT) as previously described [34], with modifications. Test samples were serially diluted 2-fold from 1:20 to 1:320. Three µl of a 10^5^ pfu/ml (~ 300 pfu) of purified vaccinia virus WR was added to 300 µl of each serum dilution. As a virus control, 3 µl of 10^5^ pfu/ml virus suspension was added to 300 µl of medium. The serum/virus mixtures and the virus control were incubated at 37^°^C for 1.5 hours, and used to infect confluent monolayers of BSC-40 cells. Cells were infected with 100 µl per well in duplicate wells per serum dilution, and also with the virus control. A pair of wells (cell control) received un-supplemented DMEM medium. After 2 hours of infection, infection medium was aspirated from all wells, and 1 ml of DMEM medium supplemented with 5% fetal bovine serum, 50 µg/ml gentamicin, and 0.5% CMC, was added to each well. The cells were incubated for an additional 24 hours, after which they were fixed/stained with crystal violet solution containing formalin. After washing off excess crystal violet stain with water, the number of plaques in each well was counted and the average numbers of plaques were determined. The percent virus neutralization in each dilution of the test serum sample was calculated using the mean plaque count in the virus control as the denominator. The 50% neutralizing titer (NT_50_) for each test serum sample was computed using the GraphPad Prism 5 software (GraphPad Software, Inc., La Jolla, CA).

### Vaccinia Extracellular Virus (EV) Neutralization

Vaccinia virus EV neutralization antibody was quantified as previously described [35] with modifications. The preparation of fresh vaccinia EV by infection of confluent monolayers of RK-13 cells with the IHD-J strain was as described [30]. Clarified infected cell culture medium supernatant (~ 2 x 10^8^ pfu/mL) was used in the EV neutralization assay in the presence or absence of 1% baby rabbit complement. In a final reaction volume of 300 µl, each test serum sample was diluted 1:50 in medium, and the 10F5 anti-L1 monoclonal antibody (purified from hybridomas obtained from Dr. Bernard Moss, NIH) and rabbit anti-A27 polyclonal antibody (a gift from Dr. Yong He, CBER/FDA) were added to final dilutions of 1:100 and 1:50, respectively. As a virus control, 3 µl of 10^5^ pfu/ml EV suspension was added to 300 µl of medium containing the anti-L1 and anti-A27 antibodies. For a second set of EV neutralization in the presence of complement, baby rabbit complement (Cedarlane Laboratories, Burlington, North Carolina) was added to a final concentration of 1%. Fresh EV was diluted to 10^5^ pfu/ml in DMEM medium, and 3 µl was added to each reaction. Following incubation at 37 ^°^C for 1.5 hours, virus/antibody mixtures were used to infect confluent monolayers of BSC-40 cells. Cells were infected with 100 µl per well in replicate per each serum dilution, and also with the virus control. Control wells received only DMEM medium. Assay plates were incubated for 24 hours, then fixed/stained with crystal violet as described above. The number of plaques in each well was counted, and the percent EV neutralization for each test sample was calculated using the mean plaque count in the virus control as the denominator.

### Statistical Analysis

For analysis of antibody titers in the various treatment groups of mouse immunization experiments, significant differences were analyzed using either an unpaired, two-tailed Student’s t test or a one-way analysis of variance (ANOVA). Fisher’s exact test was used to compare differences in the number of surviving animals in various treatment groups following challenge. In all cases, significant differences between groups were defined as P < 0.05 (InStat, GraphPad Software, Inc.).

## Results

### Construction and characterization of recombinant vaccinia viruses lacking A33, B5 or both A33 and B5 proteins

To investigate the role and importance of the extracellular envelope proteins A33 and B5 in eliciting protective immunity following vaccination, we used a gene knockout approach to generate vaccinia virus recombinants in which the *A33R*, *B5R*, or both *A33R* and *B5R* open reading frames were deleted. Although *A33R* and *B5R* single gene knockout vaccinia viruses have been reported previously, we chose to delete these genes in a vaccine-derived strain of vaccinia virus in order to evaluate the contribution of these proteins in an animal model of vaccination. At the time when these studies were initiated, the only available licensed smallpox vaccine in the United States was Dryvax, a non-clonal virus prepared by growth on the skin of calves. To facilitate the generation of gene knockout recombinants, a platform virus was developed by clonal selection after plaquing Dryvax vaccine virus on BSC-1 cells. Individual plaque isolates of Dryvax were characterized in vitro by viral genome analysis and replication in tissue culture, and in vivo for their ability to elicit a protective immune response in mice (Supporting Figure S1). One of the clones, DV-3, that showed a similar growth kinetics to Dryvax in vitro and elicited a protective immune response in a mouse intranasal challenge with the Western Reserve strain (WR) of vaccinia virus (data not shown), was selected as a platform for the construction of recombinant viruses A33Rko (lacking *A33R*); B5Rko (lacking *B5R*); and A33R/B5Rko (a double knockout lacking both *A33R* and *B5R*).

A33Rko and B5Rko were constructed by homologous recombination of plasmid vectors containing the sequence of the green fluorescent protein (GFP) and flanking sequences of the either target gene with DV-3. The A33R/B5Rko double knockout was constructed by using the A33Rko virus for the insertion of the DsRed gene at the *B5R* gene locus. The recombinants were plaque-purified and stock viruses were prepared from infected BSC-1 cells, and purified on 36% sucrose cushion. In order to verify the deletion of *A33R* and *B5R* or both genes from A33Rko, B5Rko and A33R/B5Rko, respectively, we extracted DNA from the recombinant viruses and DV-3, and used internal primers for the *A33R* and *B5R* genes in a PCR assay to amplify co-ordinates 12 to 348 (337 base pairs) and co-ordinates 130 to 664 (535 base pairs) of *A33R* and *B5R*, respectively. As a control, the entire sequence of the 792 base pairs of the *C3L* open reading frame was amplified from the three recombinants, and DV-3. The results showed that whereas *C3L* was amplified from all recombinant viruses and DV-3, and that the *A33R* and *B5R* sequences could be amplified from DV-3, the deleted genes could not be amplified from their respective knockout, confirming the absence of A33R from A33Rko, B5R from B5Rko, and the absence of both genes (*A33R* and *B5R*) from A33R/B5Rko (data not shown).

**Figure 1:**
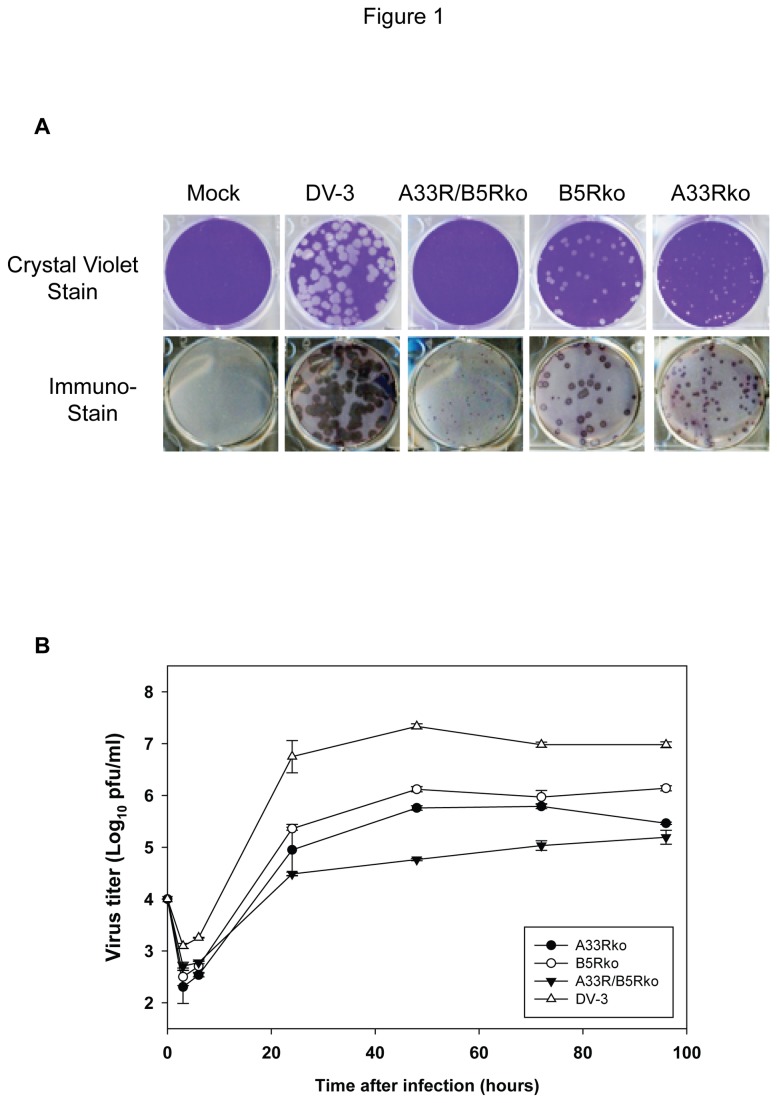
Characterization of vaccinia virus recombinants with EV gene deletions. DV-3 and recombinant vaccinia virus carrying deletions of the *A33R* gene (A33Rko), the *B5R* gene (B5Rko), or both *A33R* and *B5R* (A33Rko/B5Rko) were characterized for plaque formation and virus replication. (A) Plaque formation of viruses in BSC-40 cells after 7 days of incubation using both crystal violet staining and immunostaining methods. (B) Virus replication in BSC-40 cells. Monolayers were infected at a multiplicity of infection of 0.01 with DV-3 or each of the recombinant knockout viruses and total virus titer was determined by plaque assay at the indicated times after infection.

The recombinants were characterized for growth in cell culture. Confluent monolayers of BSC-40 cells were infected with A33Rko, B5Rko, A33/B5Rko or DV-3, and cells were stained with crystal violet after 7 days of incubation in order to detect virus plaques (Figure 1A, top panels). Viruses A33Rko, B5Rko and DV-3 formed visible plaques, though the plaque sizes of the A33Rko and B5Rko were relatively smaller in size than DV-3 and were in the size order A33Rko < B5Rko < DV-3. The A33R/B5Rko virus did not form visible plaques even after 7 days of incubation. All viruses, however, formed detectable plaques when visualized by immunostaining after 7 days of incubation, with a plaque size order that was consistent with that observed by crystal violet staining. The A33R/B5Rko virus formed small infection foci that were detectable and quantifiable only by immunostaining (Figure 1A, lower panels). In addition, the double knockout recombinant virus did not produce visible plaques in DF-1, RK-13, BHK-21 and NIH 3T3 cell lines (data not shown).

To determine the growth characteristics of the recombinant virus constructs, BSC-40 cell monolayers were infected with DV-3 or with the individual recombinant knockout viruses at a multiplicity of infection of 0.01. Infected cells were harvested after 3, 6, 24, 48, 72, and 96 hours post-infection, lysed, and the virus titer in each lysate was determined by plaque assay with crystal violet staining (A33Rko, B5Rko, and DV-3) or by immunostaining (A33R/B5Rko) (Figure 1B). In preliminary experiments, titers of replication-competent vaccinia virus determined by crystal violet staining and by immunostaining were equivalent. While the near-maximal peak titer for DV-3 was attained by 24 hours, the titer for the recombinant viruses seemed to peak at 48 hours (A33Rko and B5Rko) or later (A33R/B5Rko). The peak titer of DV-3 (7.3 log_10_) was about 1.5, 1.2, and 2.1 log_10_ higher than the peak titers for A33Rko, B5Rko, and A33R/B5Rko, respectively.

### Antibody response to EV knockout viruses and vaccinia virus neutralization

**Figure 2:**
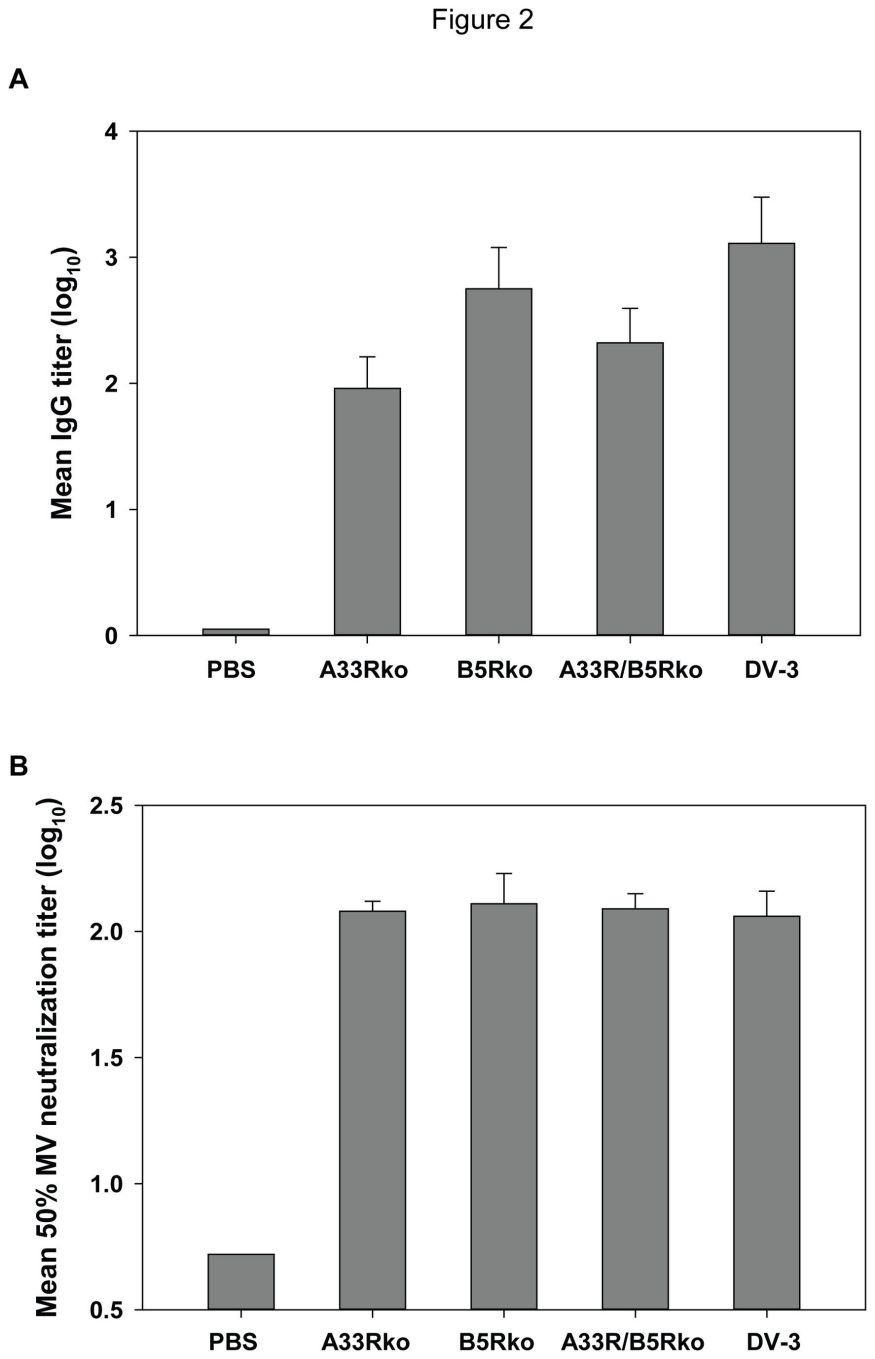
Antibody response to EV knockout viruses. Groups of mice (5 mice per group) were inoculated by tail scarification with 10^5^ pfu of recombinant viruses A33Rko, B5Rko, A33R/B5Rko, or the parent, DV-3. Immune sera obtained 3 weeks after vaccination were tested in vitro for vaccinia-specific IgG by ELISA (A) and the neutralization of ~ 300 pfu of purified vaccinia virus strain WR MV (B). Data represent mean IgG titer (A) or mean neutralizing titer (B), and error bars represent standard deviation.

Data from the characterization of the recombinant viruses versus DV-3 suggested that differences in replication kinetics might result in differences in the immune response elicited by these virus constructs. Thus, we next investigated the effect of the deletions of *A33R*, *B5R* or both genes on the antibody response to vaccinia virus. Groups of mice were vaccinated with equivalent doses (10^5^ pfu per mouse) of A33Rko, B5Rko, A33R/B5Rko, or DV-3, or were mock-immunized with diluent (PBS) via tail scarification. Three weeks post immunization, mouse sera were collected and tested for the presence of vaccinia virus-specific IgG by ELISA using inactivated Dryvax as capture antigen. Except for the PBS-treated group that had no detectable vaccinia-specific IgG, antisera from mice immunized with A33Rko, B5Rko, A33R/B5Rko, or DV-3, had measurable levels of IgG (Figure 2A). The sera from the DV-3 immunized group had a significantly higher total vaccinia-specific IgG titer than sera from either the A33Rko or A33R/B5Rko immunized groups of animals.

Vaccinia MV-neutralizing antibody in the antisera of the different treatment groups was determined by standard plaque-reduction neutralization test (PRNT). Somewhat surprisingly, the levels of neutralizing antibody (Figure 2B) was similar in all treatment groups (except for the PBS group that had no neutralizing antibody), in spite of the measured differences in the levels of total IgG. When mice were immunized with lower doses of DV-3 and the double EV knockout A33R/B5Rko (e.g., 10^3^ or 10^4^ pfu), the NT_50_ were not significantly above those in control sera, likely reflecting the limits of assay sensitivity.

### Antibody response to EV proteins and neutralization of vaccinia EV

**Figure 3:**
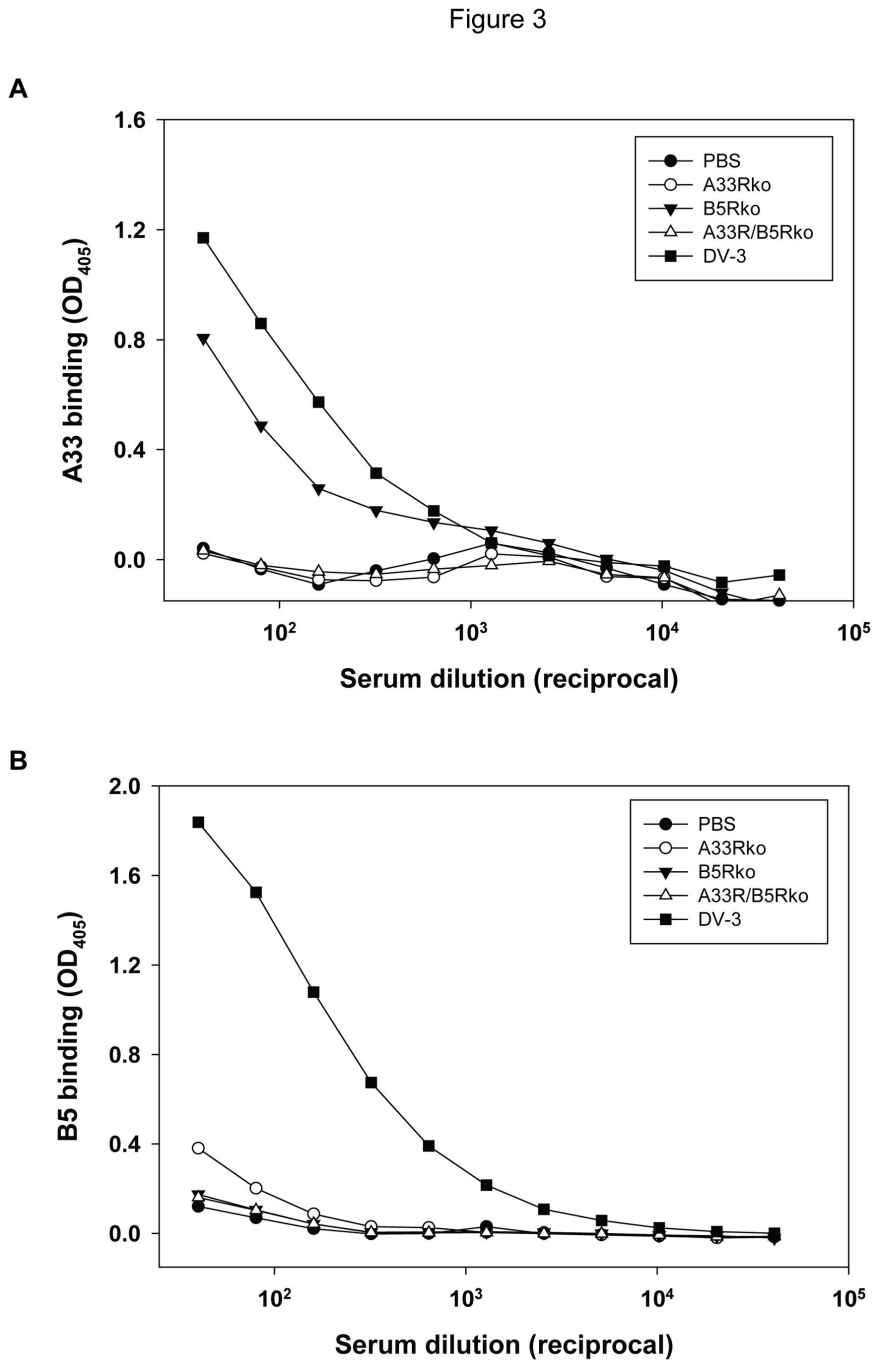
EV-specific antibody response to EV knockout viruses. Immune sera were obtained 3 weeks after vaccination with A33Rko, B5Rko, A33R/B5Rko, or DV-3 and were tested for A33-specific IgG (A) and B5-specific IgG (B) by ELISA.

Antisera from mice vaccinated with DV-3 or the EV knockout viruses were analyzed for the presence of antibodies to the EV proteins A33 and B5 by ELISA, using affinity-purified A33 or B5 protein (Figure 3). As expected, antisera from the DV-3 immunization group had easily quantifiable levels of IgG antibodies to both A33 and B5, whereas there was no detectable A33 or B5 antibody in sera from either the PBS or the A33R/B5Rko vaccinated animals. Antisera from mice vaccinated with B5Rko had no detectable B5 IgG, but A33-specifc IgG was detectable. In contrast, antisera from mice vaccinated with A33Rko did not have detectable A33 IgG, and B5-specific IgG was only detectable at the most concentrated serum dilution tested. The results confirmed the absence of the expected EV protein in each recombinant knockout virus, and also indicated that deletion of *A33R* affected the antibody response to B5, possibly by affecting expression or presentation of B5.

**Figure 4:**
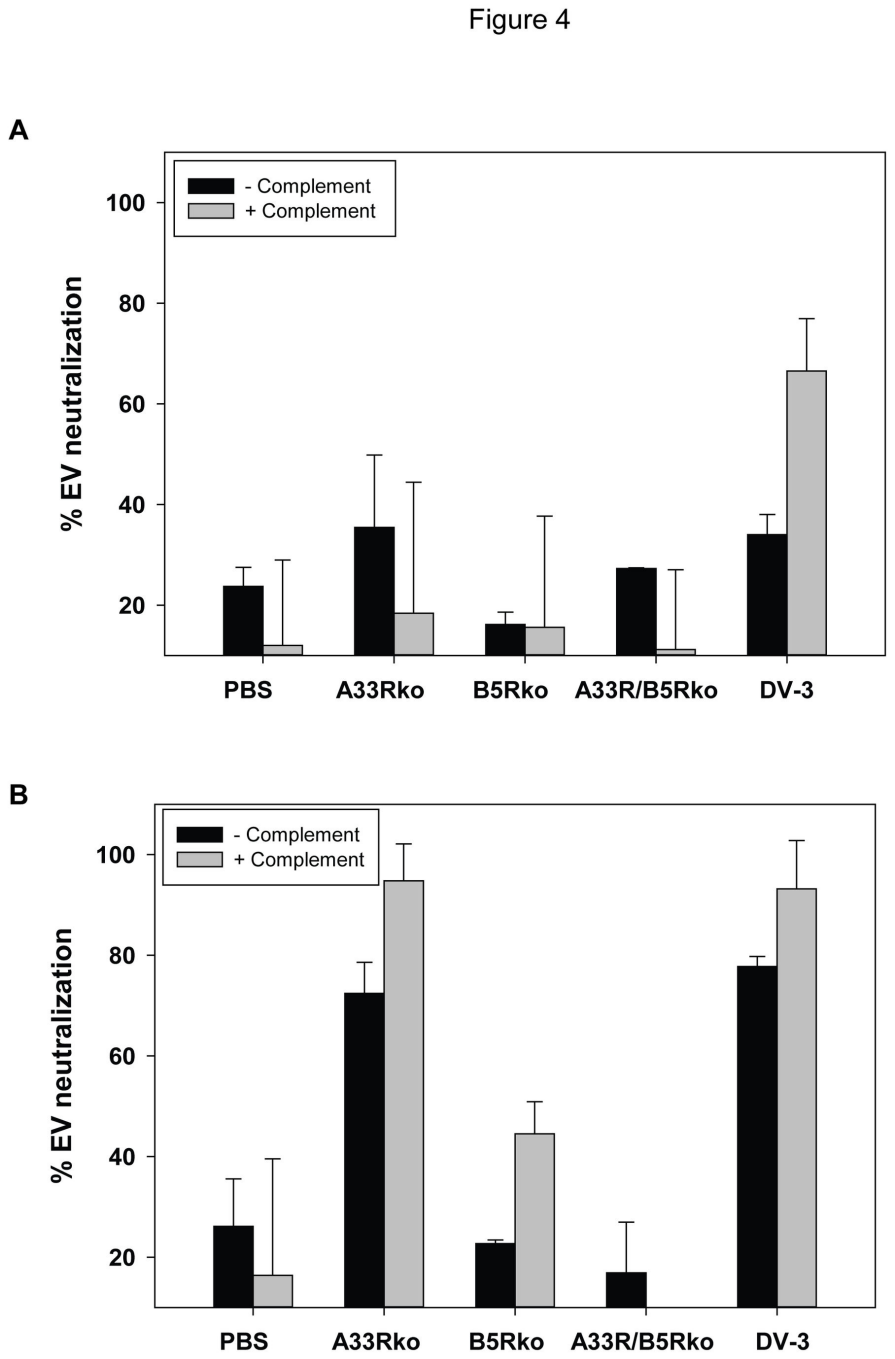
Neutralization of the EV form of vaccinia virus. Pooled immune sera (from groups of 5 mice) were obtained from mice 3 weeks after a single inoculation (A) or 3 weeks after 3 inoculations (3 weeks between inoculations) with recombinant A33Rko, B5Rko, A33R/B5Rko viruses or DV-3, were tested for the neutralization of ~ 300 pfu of the EV form of vaccinia virus at a dilution of 1:50 of each test serum pool, in the presence or absence of baby rabbit complement.

Although traditional PRNT assays measure the neutralization of the MV form of vaccinia virus, modified assays have been developed that specifically measure neutralization of the EV form of the virus [35]. In order to test EV-neutralizing activity in antisera obtained from mice that had been vaccinated with DV-3 or with the EV knockout viruses A33Rko, B5Rko, or A33R/B5Rko, we used freshly-prepared EV and protocol as previously described [30]. In this assay, undiluted antisera obtained from animals following a single immunization by tail scarification with DV-3 or the EV knockout viruses weakly neutralized vaccinia EV (Figure 4A). Only about 35% of the input EV could be neutralized by antisera from either DV-3 or A33Rko immunized mice, although with neutralization in the presence of complement, DV-3 neutralization of EV increased to approximately 65%. In an attempt to amplify the EV antibody response to DV-3 and the EV knockout viruses, the mice in each group were boosted with 2 additional immunizations with each virus. Undiluted antisera obtained three weeks after the third immunization were tested for EV-neutralizing activity in the presence or absence of complement (Figure 4B). Antisera from both the A33Rko and DV-3 showed measurable EV-neutralizing activity of 72% and 78%, respectively, and this activity was further enhanced in the presence of complement. EV neutralization with B5Rko and A33R/B5Rko was still not above background (antisera from PBS immunized mice) even after 3 virus immunizations, although B5Rko antisera neutralized approximately 40% of the input EV in the presence of complement. The results from these experiments indicate that EV-specific neutralizing activity is difficult to detect in the sera of mice immunized with DV-3 or EV knockout viruses, possibly due to either the sensitivity of the assay or the absence of sufficient levels of EV neutralizing antibody. Nevertheless, the results confirm that deletion of *B5R* in both B5Rko and A33R/B5Rko results in a loss of EV antibody following vaccination of mice with these viruses.

### Lethal intranasal vaccinia virus challenge of DV-3 and A33R/B5Rko vaccinated mice

The effect of deletion of vaccinia virus genes encoding the EV proteins A33 and/or B5 on the protection afforded by vaccination was evaluated by intranasal challenge with a lethal dose of either vaccinia virus strains WR or IHD-J. Although the neuro-adapted WR strain is commonly used as a challenge virus in mouse models of orthopoxvirus vaccination, the IHD-J strain was of interest because of its ability to produce relatively higher amounts of released EV form of vaccinia virus than the WR strain. Although the LD_50_ determined for WR was lower than that of IHD-J in BALB/c mice (4 x 10^3^ versus 4 x 10^4^, respectively), both viruses were sufficiently pathogenic in the intranasal challenge model to allow a range of challenge doses to be evaluated. Additional characterization of the 2 challenge viruses revealed that the IHD-J strain exhibited more extensive comet formation in tissue culture using liquid overlay and had a higher relative proportion of released EV to MV virus than WR (data not shown).

In initial experiments to evaluate the protective effect afforded by DV-3 and the EV knockout derivatives, mice immunized with 10^5^ pfu of either DV-3 or any of the EV knockout virus recombinants A33Rko, B5Rko or A33R/B5Rko were protected from a subsequent challenge with 25 LD_50_ of WR, whereas no mice in the control PBS-immunized group survived this challenge (data for A33Rko and B5Rko not shown; data for A33R/B5Rko included in Tables 1 and 2). Further, 10^5^ pfu of DV-3 or the double EV knockout virus A33R/B5Rko fully protected mice from a similar challenge of 25 LD_50_ of IHD-J. These results suggested that the absence of EV proteins A33 and/or B5 did not dramatically reduce the ability of the vaccinia vaccine virus to provide protection in the intranasal challenge model. Consequently, additional experiments using different immunization doses and challenge doses were designed in order to determine whether deletion of the EV-encoding genes *A33R* and *B5R* might have a more measurable effect on the protective capacity of the vaccinia virus used for immunization. Animals challenged with a lethal dose of virus were assessed for morbidity by weight loss and mortality resulting from virus challenge.

**Table 1:** Protective effect of DV-3 and A33R/B5Rko vaccination against intranasal challenge with vaccinia virus WR.

		**Challenge**		**Deaths^a^**		**Mean time to Death^b^**		**Max Weight Loss^c^**	
**Immunization**									
PBS		WR -25 LD_50_		25/25 (4)		6.6±1.3		25.0% (d7)
10^3^ DV-3		WR -25 LD_50_		9/15 (2)		6.7±0.7		19.7% (d6)
10^3^ A33R/B5Rko		WR -25 LD_50_		12/15 (2)		6.6±0.7		22.9% (d7)
10^3^ DV-3		WR -100 LD_50_		5/5 (1)		6.0±0.7		25.0% (d7)
10^3^ A33R/B5Rko		WR -100 LD_50_		4/5 (1)		6.6±1.0		24.9% (d7)
10^4^ DV-3		WR -25 LD_50_		2/10 (2)		7.5±2.1		17.5% (d5)
10^4^ A33R/B5Rko		WR -25 LD_50_		5/10 (2)		7.2±0.4		17.7% (d6)
10^4^ DV-3		WR -100 LD_50_		2/5 (1)		7.5±0.7		22.1% (d7)
10^4^ A33R/B5Rko		WR -100 LD_50_		4/5 (1)		6.3±0.5		20.5% (d6)
10^5^ DV-3		WR -25 LD_50_		0/20 (3)		0		7.9% (d3)
10^5^ A33R/B5Rko		WR -25 LD_50_		1/20 (3)		7.0±0		13.0% (d5)
10^5^ DV-3		WR -100 LD_50_		0/5 (1)		0		19.8% (d4)
10^5^ A33R/B5Rko		WR -100 LD_50_		1/5 (1)		8.0±0		18.3% (d7)

a Combined results from the number of independent challenge experiments in ( )

b Mean time to death in days ± SD

c Maximum mean weight loss and day of occurrence

**Table 2:** Protective effect of DV-3 and A33R/B5Rko vaccination against intranasal challenge with vaccinia virus IHD-J.

		**Challenge**		**Deaths^a^**		**Mean time to Death^b^**		**Max Weight Loss^c^**	
**Immunization**									
PBS		IHD-J -25 LD_50_		15/15 (3)		6.1±0.8		25.0% (d7)
10^3^ DV-3		IHD-J -25 LD_50_		10/14 (2)		6.4±0.8		21.5% (d7)
10^3^ A33R/B5Rko		IHD-J -25 LD_50_		6/15 (2)		7.0±1.3		17.7% (d7)
10^3^ DV-3		IHD-J -100 LD_50_		4/5 (1)		6.0±1.4		24.0% (d7)
10^3^ A33R/B5Rko		IHD-J -100 LD_50_		4/5 (1)		6.3±1.5		22.7% (d7)
10^4^ DV-3		IHD-J -25 LD_50_		2/5 (1)		8.0±1.4		17.9% (d7)
10^4^ A33R/B5Rko		IHD-J -25 LD_50_		0/5 (1)		0		15.9% (d6)
10^5^ DV-3		IHD-J -25 LD_50_		0/15 (2)		0		6.5% (d4)
10^5^ A33R/B5Rko		IHD-J -25 LD_50_		0/15 (2)		0		7.3% (d5)
10^5^ DV-3		IHD-J -100 LD_50_		0/5 (1)		0		14.6% (d6)
10^5^ A33R/B5Rko		IHD-J -100 LD_50_		1/5 (1)		0		8.1% (d6)

a Combined results from the number of independent challenge experiments in ( )

b Mean time to death in days ± SD

c Maximum mean weight loss and day of occurrence

**Figure 5:**
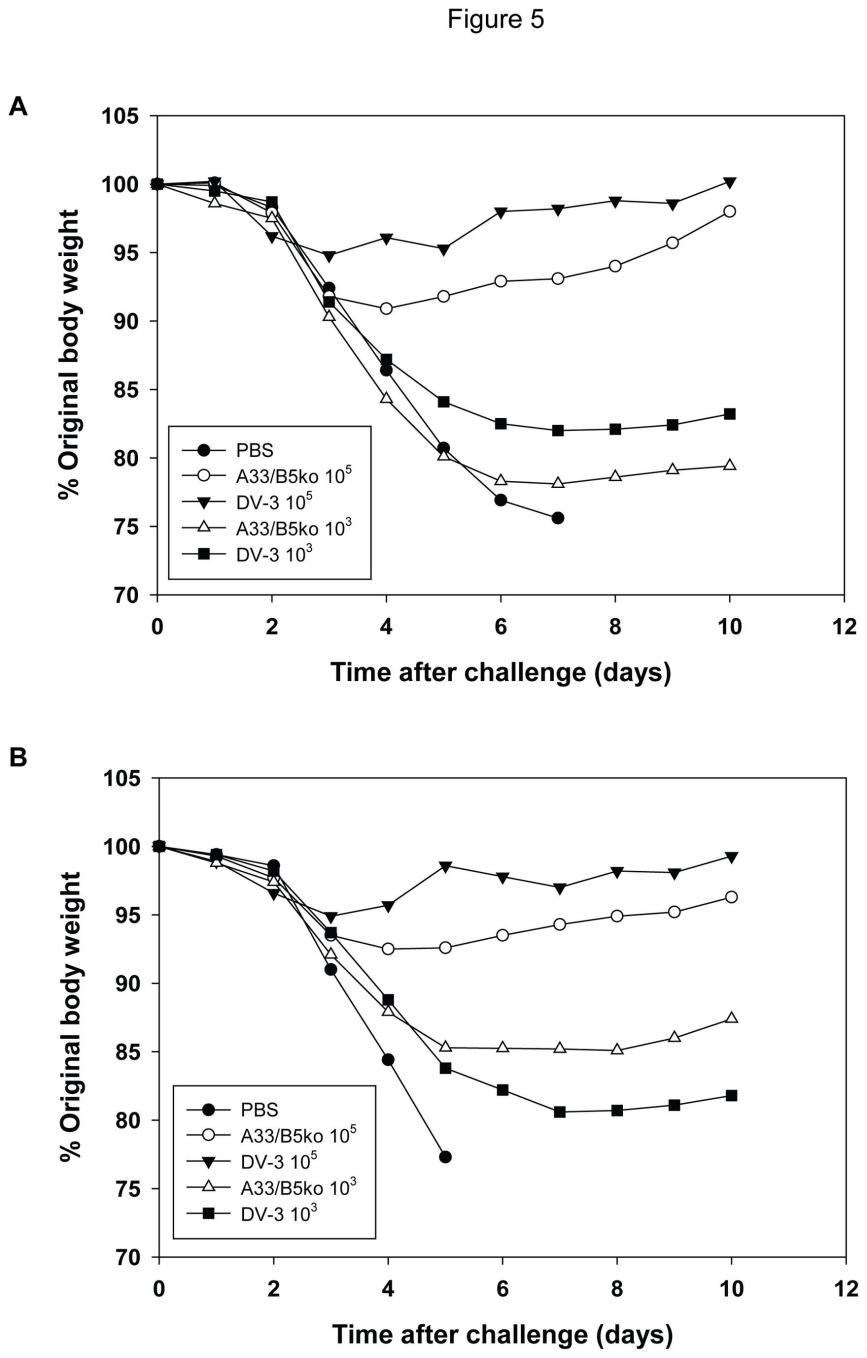
Protection of mice from vaccinia WR or IHD-J challenge. Mice (groups of 5) were mock-immunized with PBS or immunized with either 10^3^ or 10^5^ pfu of A33R/B5Rko virus or DV-3. Mice were challenged intranasally with 25 LD_50_ of either vaccinia WR (A) or IHD-J (B) and were observed for 10 days for weight loss and lethality. The mean percentage change in body weight in each group is shown. Data are representative of two identical challenge experiments. The results of all challenge experiments are presented in Tables 1 and 2.

Figure 5 shows the weight loss curves for a representative experiment in which mice vaccinated with DV-3 or A33R/B5Rko at either a 10^3^ or 10^5^ pfu dose and challenged with 25 LD_50_ of either vaccinia WR (Figure 5A) or IHD-J (Figure 5B). While there was a modest weight loss in animals receiving a vaccine dose of 10^5^ pfu, substantially more weight loss was observed in animals immunized with 10^3^ pfu of either vaccine. Regardless of the challenge dose, however, there was no significant difference between the mean weight loss exhibited by animals receiving DV-3 and A33R/B5Rko (error bars not shown). Tables 1 and 2 summarize a series of experiments in which mice were immunized by scarification with doses of DV-3 or A33R/B5Rko from 10^3^ to 10^5^ pfu and subsequently challenged intranasally with 25 or 100 LD_50_s of vaccinia virus WR (Table 1) or IHD-J (Table 2). In each individual experiment, mice in the PBS treatment group received 25 LD_50_ of the challenge virus. No mouse in the PBS treatment groups in any experiment survived. All mice vaccinated with 10^5^ pfu of A33R/B5Rko or DV-3 survived subsequent intranasal challenge with 25 or 100 LD_50_s of WR or IHD-J, except for a single mouse in one experiment that was immunized with A33R/B5Rko and challenged with 100 LD_50_ of WR. Whereas the higher immunization dose afforded virtually complete protection against both challenge doses with either WR or IHD-J, low dose vaccination with 10^3^ pfu of either A33R/B5Rko or DV-3 provided only partial protection against challenge. For example, 3/15 and six-fifteenths of A33R/B5Rko and DV-3 immunized mice, respectively, survived a 25 LD_50_ challenge with WR; 9/15 and four-fifteenths of A33R/B5Rko and DV-3 immunized mice, respectively, survived a 25 LD_50_ of IHD-J. At the low dose immunization of 10^3^ pfu, poor protection was observed upon a challenge with 100 LD_50_. Only 1 of 5 animals immunized with either A33R/B5Rko or DV-3 survived a 100 LD_50_ challenge with IHD-J; 1/5 and 0/5 mice immunized with A33R/B5Rko or DV-3, respectively, survived a 100 LD_50_ challenge with WR. None of the observed differences in survival between the DV-3 and A33R/B5Rko immunized groups were significant for either challenge virus at any dose. Further, the mean time to death for animals who succumbed to challenge and the mean maximum weight loss resulting from challenge was similar for both DV-3 and A33R/B5Rko vaccinated groups.

Although fewer experiments were performed with an immunization dose of 10^4^ pfu, the protection afforded by vaccination with either A33R/B5Rko or DV-3 appeared to be intermediate between the 10^3^ and 10^5^ immunization doses. For example, 5/10 (A33/B5Rko) and 8/10 (DV-3) survived 25 LD_50_ of WR. At the 100 LD_50_ challenge dose, 1/5 of mice vaccinated with 10^4^ pfu of A33R/B5Rko and three-fifths of those vaccinated with the same dose of DV-3 survived a 100 LD_50_ challenge with WR (Table 1). Similarly, 5/5 and three-fifths of A33/B5Rko and DV-3 treated mice, respectively, survived a 25 LD_50_ challenge with IHD-J (Table 2).

**Figure 6:**
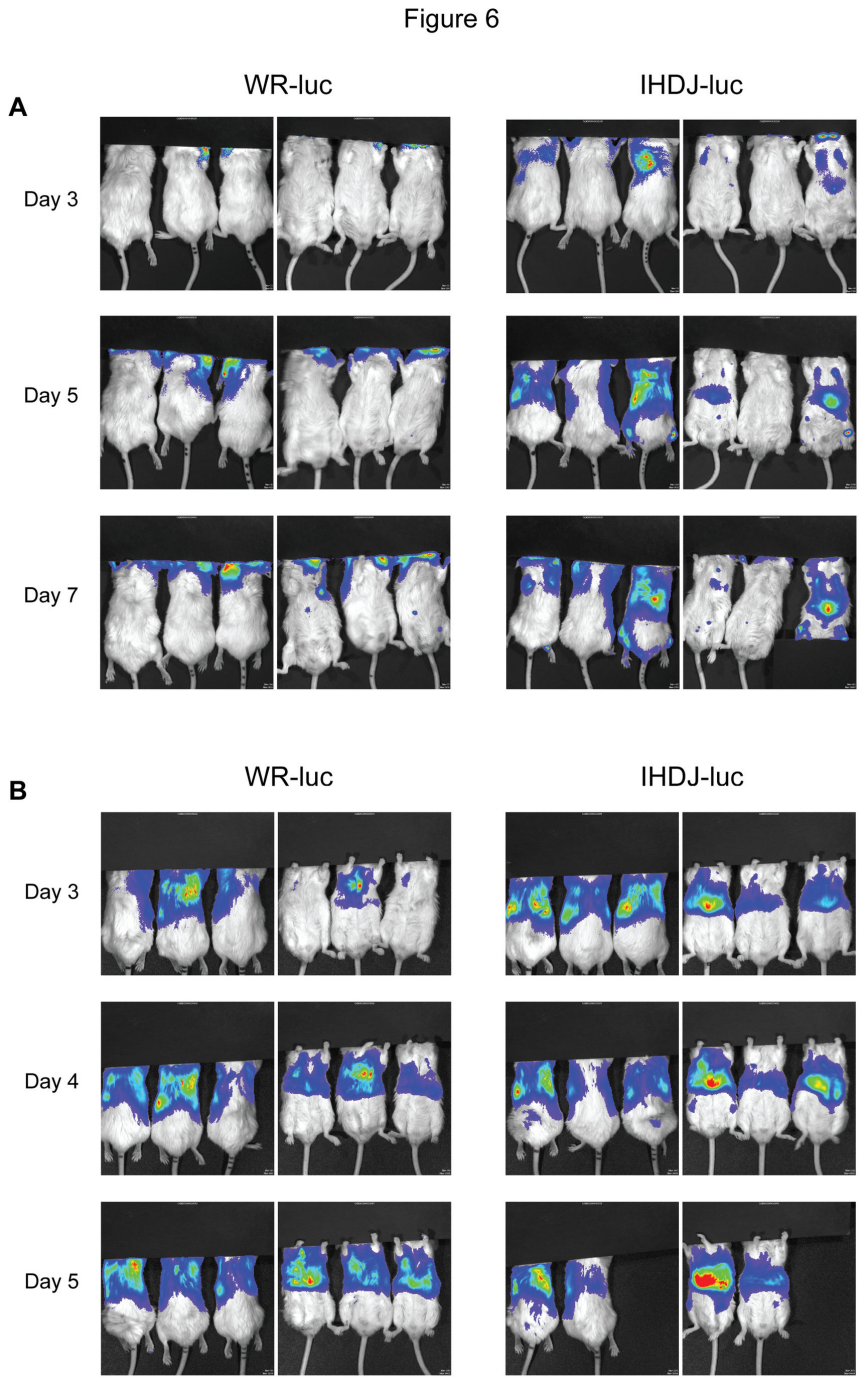
In vivo dissemination of WR-Luc and IHD-J-Luc vaccinia viruses. Mice were inoculated with either 10 LD_50_ (A) or 100 LD_50_ (B) of either WR-luc or IHDJ-luc and virus dissemination was recorded by in vivo imaging on days 3, 5, and 7 (A) or days 3, 4, and 5 (B).

Since vaccination with the A33R/B5Rko virus appeared to be able to protect mice as well as DV-3 from a lethal intranasal challenge with vaccinia virus, we further compared the ability of the 2 vaccine viruses to affect disease progression following challenge. In order to allow for the monitoring of disease progression in real time, using virus dissemination as a surrogate marker, we constructed WR and IHD-J recombinants expressing luciferase, WR-luc and IHDJ-luc, respectively, in which the luciferase gene was inserted at the locus of the homolog of the cowpoxvirus host-range gene, *CP77*, which is truncated in vaccinia virus. The recombinant viruses WR-luc and IHDJ-luc had similar LD_50_ values (4.2 x 10^4^ and 3.2 x 10^4^, respectively), and thus were pathogenic in mice with the added advantage that virus dissemination could be monitored in vivo in real time. Similar to the IHD-J and WR parent viruses, IHDJ-luc exhibited more extensive comet formation in tissue culture using liquid overlay and had a higher relative proportion of released EV to MV virus than WR-luc. In experiments designed to compare in vivo spread of the 2 luciferase-expressing viruses, IHDJ-luc appeared to disseminate faster than WR-luc following intranasal inoculation (data not shown). Naive mice were inoculated with either 10 LD_50_ or 100 LD_50_ of either WR-luc or IHDJ-luc and virus dissemination was recorded by in vivo imaging on days 3 through 7 (Figure 6). At the 10 LD_50_ challenge dose, there appeared to be a faster and more extensive spread of the IHDJ-luc virus at each tested timepoint (Figure 6A) as captured by in vivo imaging of the luciferase signal. At the 100 LD_50_ challenge dose (Figure 6B) however, both viruses disseminated rapidly throughout the animal bodies, with a somewhat more pronounced luciferase dissemination at day 3 in the IHDJ-luc infected animals. The results suggested that the IHDJ-luc virus might be useful for discerning subtle differences in challenge virus spread resulting from the deletion of EV antigens in the vaccine virus.

**Figure 7:**
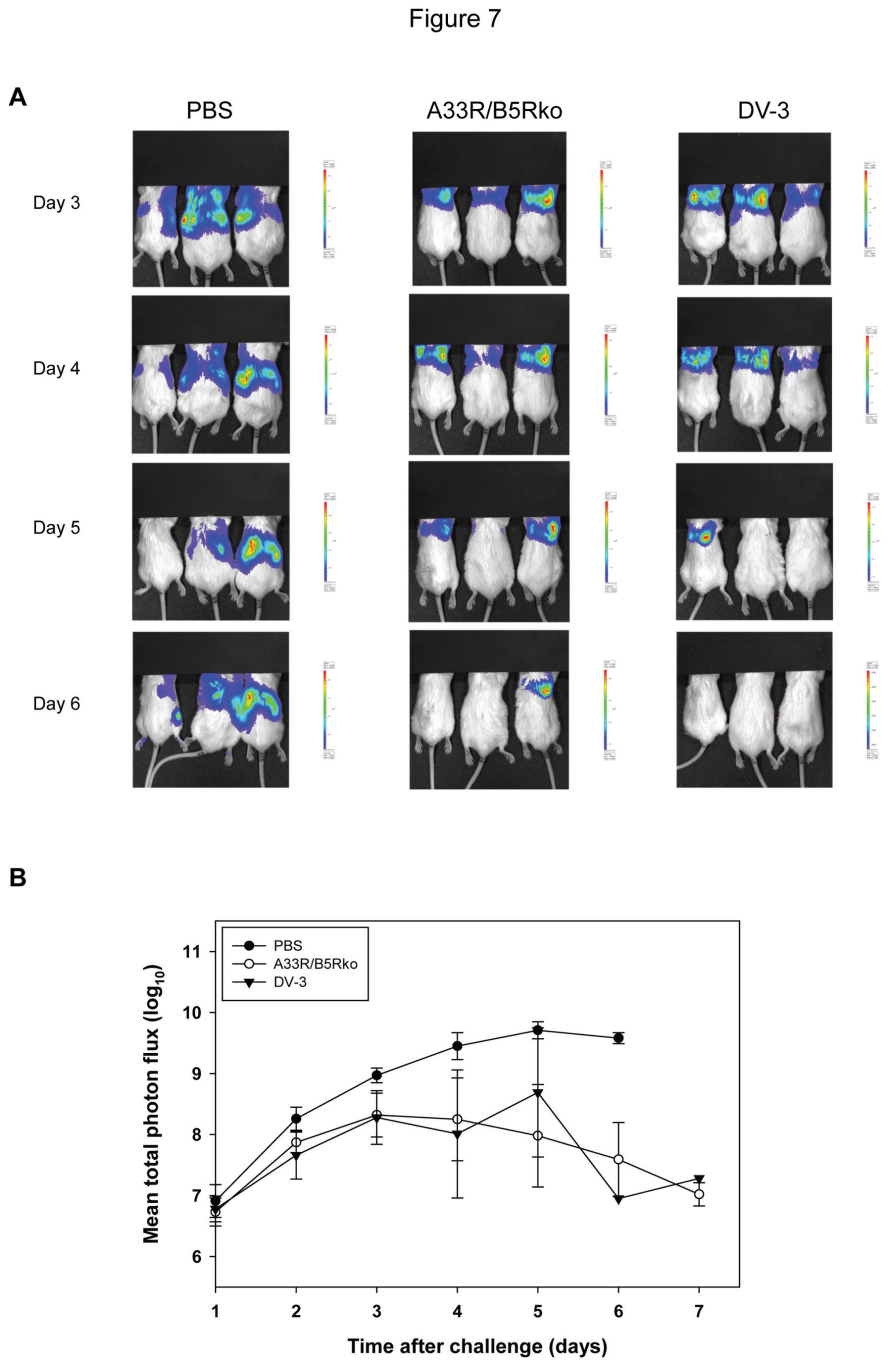
Effect of vaccination on real-time dissemination of IHDJ-luc challenge. Groups of mice were vaccinated with A33R/B5Rko, DV-3, or mock vaccinated with PBS and challenged with 25 LD_50_ of IHDJ-luc. Mice were observed and weighed daily for 10 days post-challenge, and all mice in each group were imaged in two sets on days 1 to 7 post-challenge. (A) Representative in vivo imaging of mice from each group on days 3, 4, 5, and 6 post-challenge. (B) Mean total photon flux in each group of vaccinated and challenged mice.

To test the effect of *A33R* and *B5R* deletions in the A33R/B5Rko vaccine virus on the real-time dissemination of an IHDJ-luc challenge virus, groups of 5 mice were vaccinated with 10^3^ pfu of either A33R/B5Rko or DV-3 and challenged with 25 LD_50_ of IHDJ-luc, 3 weeks after immunization. Mice were observed and weighed daily for 10 days post-challenge, and each group was imaged in sets of three on days 1 to 7 post-challenge. Images of the first set of 3 mice in each group are shown (Figure 7A), and are representative of each group. Mice in the PBS-treated group exhibited extensive virus spread from day 3 through day 6 post-challenge with a mean total photon flux of 5.4 x 10^9^ as the luciferase signal peaked on day 5 (Figure 7B). All of the mice in this group eventually died. All mice in the A33R/B5Rko immunized group survived, as did four-fifths of the DV-3 immunized group. In both groups of vaccinated mice, imaging revealed some virus dissemination on days 3 and 4, although not to the extent of that observed in the PBS-treated mice. The luciferase signal in both vaccinated groups began resolving on days 5 and 6 (Figure 7A). There was no significant difference in the mean total photon flux in the A33R/B5Rko-immunized group compared to the DV-3 immunized group (Figure 7B). In a similarly designed experiment, mice were immunized with 10^5^ pfu of either A33R/B5Rko or DV-3 and challenged with 100 LD_50_ of IHDJ-luc. Again, there was some virus dissemination on day 3 post-challenge even in the 2 vaccinated groups but the luciferase signal in these groups began to resolve by day 5 (data not shown). Taken together, the results from multiple experiments measuring mortality, morbidity, and virus dissemination indicated that deletion of the *A33R* and *B5R* genes appeared to have little effect on the ability of a vaccinia virus vaccine to provide protection against a lethal intranasal challenge in a mouse model.

## Discussion

A number of virus antigens and epitopes have been identified as targets for the cellular and humoral immune response against vaccinia virus smallpox vaccine [36,37]. However, it is not clear whether the immune response against these proteins and epitopes is absolutely required for the elicitation of protective immunity, nor is it clear what the relative contribution of the immune response to any particular antigen or epitope is in the overall protective response. For example, neutralizing antibodies are elicited upon vaccination to numerous MV proteins including the immunodominant H3 protein. However, if antibody to H3 is removed there is no significant reduction in the total MV-neutralizing activity, suggesting that the MV-neutralizing antibody response to vaccinia virus is highly redundant [38]. In addition, studies have underscored the importance of cellular immunity in the protection against poxvirus infections in different animal models [39,40]. Further complicating the understanding of the protective immune response to smallpox vaccination is the presence of the second form of vaccinia virus, the enveloped virion (EV), that is generated during infection and that expresses a different set of envelope proteins than the MV form of virus. The EV form of vaccinia virus plays an important role in virus pathogenesis by facilitating virus dissemination in an infected host [15], and several early reports indicated that the immune response to EV proteins forms an important component of the protective response elicited by smallpox vaccines [41,42]. More recently, studies of candidate subunit smallpox vaccines showed that animals immunized using subunits containing both MV and EV protein-expressing vectors or purified proteins were better protected than when the MV or EV components were used alone [19,20]. The EV antigen component of the subunit vaccines in most of these studies have been A33 and/or B5. Most of the EV-neutralizing activity following smallpox vaccination is directed to B5 [18,43], but antibody to A33, in the presence of complement, lyses the EV and exposes the MV to neutralizing antibody [44]. Thus, the A33 and B5 proteins are, arguably, the most prominent immune targets with respect to the neutralization of the EV form of vaccinia virus. Nevertheless, it is not clear if the antibody response to the A33 and B5 is absolutely required for the protection afforded by vaccination with smallpox vaccines. For example, the candidate smallpox vaccine LC16m8, which does not express an intact B5 protein, still provides protection in animal models of orthopoxvirus challenge [28,45,46]. Consequently, we were interested in determining the effect on protective immunity resulting from deletion of both the *A33R* and *B5R* genes from a vaccinia vaccine virus, and particularly whether the immune response to A33 and/or B5 is required sine qua non for protection against disease.

Our approach to evaluating the effect of A33 and B5 on vaccine effectiveness was to construct knockout vaccinia viruses missing either or both of these EV protein-encoding genes. Knockout viruses lacking A33 [47] and B5 [48,49] have been described previously. Those knockout viruses, which exhibited a small plaque phenotype and in the case of *B5R* deletion reduced virulence, were constructed on virus backbones such as WR and IHD-J that are relatively lethal in mice. Since our goal was to evaluate the role of specific EV antigens in vaccine-induced immunity and protection in a mouse challenge model, we chose to make EV gene knockouts in a virus strain more relevant to licensed smallpox vaccines. Toward that end, we isolated cloned viruses from the licensed smallpox vaccine Dryvax and characterized them for growth properties, immunogenicity, and protective capacity, in order to obtain a platform virus that was similar to Dryvax in certain key attributes. A somewhat similar approach was used to derive the second-generation smallpox vaccine ACAM2000 that is currently licensed for production in cell culture [50]. Although our platform virus, designated DV-3, was not characterized to the same extent as required for the licensed vaccine virus, it has similar growth properties and elicited similar antibody and protective responses in mice as the Dryvax from which it was derived. We previously used DV-3 to generate a virus deleted for the vaccinia complement control protein [24], and in this study we used DV-3 to generate recombinant vaccinia viruses lacking specific EV genes. Thus, DV-3 serves as a suitable vaccine-like virus for evaluating the role of specific virus antigens in animal models of vaccination.

A panel of DV-3 knockout viruses with deletions in individual EV genes were constructed for evaluating the contributions of EV proteins to the protective response elicited by vaccination. Viruses included A33Rko and B5Rko, which lacked the *A33R* and *B5R* genes, respectively, as well as a third recombinant, A33R/B5Rko, lacking both *A33R* and *B5R*. Although we did not directly evaluate the virulence of these EV knockout viruses in animals, it is not likely that the deletions would make them more virulent, especially as they exhibited an attenuated growth in cell culture, as reflected in their smaller plaque phenotypes (A33Rko and B5Rko) or inability to form visible plaques (A33R/B5Rko). These knockout viruses are the focus of the immunization and protection studies described in this report. However, we also generated *A34R* and *A56R* knockout viruses in DV-3 (data not shown). We observed that recombinant vaccinia viruses deleted of the *A33R* or *B5R* genes displayed relatively small plaque sizes compared to the parent virus, as did the A34Rko but not the A56Rko (data not shown), consistent with previous reports [47-49,51]. Of particular note is that the double EV-knockout A33R/B5Rko did not form visible plaques even after incubation for 1 week, and could only be detected by immunostaining (Figure 1A). In addition, this virus did not form visible plaques in other cell lines tested, including non-human primate (BSC-1 and BSC-40), murine (NIH3T3), rabbit (RK13), avian (DF-1), and hamster (BHK-21) cell lines (data not shown). A34Rko and A56Rko replicated to lower titers than Dryvax in BSC-40 cells (data not shown) and the A33R/B5Rko double knockout virus replicated even more poorly than the single EV knockout viruses (Figure 1B). Thus, while the deletion of *A33R* or *B5R* resulted in reduction in virus replication, the deletion of both *A33R* and *B5R* genes results in an even more dramatic decrease in total virus yield in cell culture.

In all of our vaccination studies, vaccine viruses are delivered by tail scarification to closely mimic the way in which smallpox vaccines are actually administered. Despite the reduction in virus progeny in cell culture, a robust antibody response to the EV knockout viruses was elicited following vaccination with 10^5^ pfu of virus. Analysis of serum samples obtained three weeks after mice had been vaccinated with 10^5^ pfu of either DV-3 or the EV knockout viruses revealed that all animals sero-converted and contained high levels of vaccinia-specific IgG as measured by ELISA using whole vaccinia virus as antigen, although the mean IgG titer in mice that were vaccinated with the parent virus DV-3 was significantly higher than those of the A33Rko or A33R/B5Rko groups (Figure 2A). In spite of the significant difference in total binding antibody levels between antisera from the A33Rko and A33R/B5Rko groups on the one hand, and the parent DV-3 on the other hand, the levels of MV-neutralizing antibody, as determined by PRNT, were similar (Figure 2B). Other studies have also noted that elimination of B5 does not have a corresponding effect on the elicited MV-neutralizing antibody [52]. The differences between the total MV-antibody determined by ELISA and MV-neutralizing antibody determined by PRNT likely reflect differences in the sensitivity of the two types of assays, as well as what the assays are measuring. Indeed, when sera was analyzed following vaccination with lower doses, neutralizing antibody could not be reliably quantified even when all animals sero-converted as measured by ELISA (data not shown).

Since the A33 and B5 proteins are constituents of the EV, we also focused on evaluating the specific antibody response to these gene products. In antigen-specific antibody binding ELISA, anti-A33 antibody response was detected in serum samples of the DV-3 and B5Rko treated animals, but not in the A33Rko or A33R/B5Rko-treated animals (Figure 3). Similarly, B5-specific IgG response was detected in the DV-3 and A33Rko antisera, but not in the B5Rko or A33/B5Rko antisera. This set of data is consistent with the absence of *A33R* or *B5R*, or both genes, in the A33Rko, B5Rko and A33R/B5Rko viruses, respectively. EV neutralization was not detectable in serum samples obtained after a single immunization with either DV-3 or the recombinant knockout viruses, although a modest level of EV neutralization was measured in the sera from DV-3 immunized animals in the presence of complement (Figure 4A). It is not known at this time whether these results reflect assay sensitivity or relatively low levels of EV neutralizing antibody following a single immunization in mice. Nevertheless, when mice were given two booster doses (at intervals of three weeks) of the respective viruses, serum samples obtained from the A33Rko and DV-3 vaccinated groups exhibited EV-neutralizing activity that was further enhanced in the presence of complement (Figure 4B). A lower but measurable level of complement-enhanced EV neutralization was also detected in the B5Rko antiserum, but this was not significantly above that in sera from PBS-immunized animals. The absence of EV-neutralizing activity in the B5Rko and A33R/B5Rko antisera can be attributed primarily to the absence of an antibody response to B5. This set of data agrees with previous studies that demonstrated that the majority of the EV-neutralizing activity in human vaccinia immunoglobulin (VIG) is directed at B5 [18], and further suggests that a robust anti-B5 response is important for the neutralization of EV in order to curtail virus spread and pathogenesis.

The absence of a robust anti-EV response to vaccination could, theoretically, enable more effective virus dissemination, and attendant pathogenesis, upon exposure to subsequent orthopoxvirus infection. Indeed, suggestions have been made that the EV antigen B5 should be included in any future smallpox vaccine and the response assessed [43]. On the other hand, licensure of new-generation smallpox vaccines will depend heavily on challenge studies in animals, and several studies on the attenuated vaccine candidate LC16m8 [30,45,46], which has a truncated B5, and related viruses missing B5 [52], have shown that B5 may be dispensable for protection against orthopoxvirus challenge. The vaccination and intranasal challenge studies reported here complement and extend those previous studies. We have eliminated B5, as well as A33 and both B5 and A33 protein expression from a strain of virus derived from the smallpox vaccine licensed in the United States and used these recombinant viruses in mouse vaccination studies that included an intranasal vaccinia virus challenge. Our studies used the mouse neuro-adapted WR strain of vaccinia virus, a commonly used virus strain for the mouse intranasal challenge model. In addition, because the work described here focused mainly on the anti-EV response, we also used the lethal IHD-J strain in our investigations since it is known to produce a relatively higher amount of released EV virions than WR. Furthermore, we constructed luciferase-expressing versions of both WR and IHD-J, WR-luc and IHDJ-luc, respectively, that retained virulence in mice, but with the added feature that challenge virus dissemination could be monitored in vivo in real time. We have previously shown the utility of these luciferase-expressing viruses for evaluating the therapeutic effect of passively administered antibodies on orthopoxvirus disease [53,54]. Thus, these viruses should serve as useful tools for evaluating the effects of either therapeutic or prophylactic treatments for orthpoxvirus infections.

In initial studies using individual EV knockout viruses, mice that were vaccinated with A33Rko, B5Rko, A34R or A56R were protected just as well as those in the DV-3 treatment group in intranasal challenge models using the WR strain or the IHD-J strain of vaccinia virus (not shown). Thus, subsequent protection and challenge experiments focused on an extensive comparison of the A33R/B5Rko double knockout with DV-3, using a range of immunization doses and challenge doses of 25 to 100 LD_50_s of either WR or IHD-J viruses. The results from numerous challenge experiments, assessing both mortality and morbidity by weight loss, indicated that deletion of EV genes from a vaccine-like virus, including both *A33R* and *B5R*, did not compromise protection afforded by vaccination. Similarly, when we used WR-luc and IHDJ-luc as challenge viruses and monitored virus spread in vivo in real time, there was little difference between the protective effect of DV-3 and the A33R/B5Rko double EV-knockout vaccine virus. Both viruses protected animals from death and appeared to clear challenge virus at approximately the same rate. In summary, the results from all of the animal challenge experiments, using mortality, morbidity, and virus dissemination as measures of protection, indicated that deletion of both EV genes *A33R* and *B5R* had little effect on the ability of a vaccine virus to provide protection against a lethal intranasal vaccinia virus challenge in a mouse model. Although we did not evaluate the differences in the cell-mediated immune responses elicited by the recombinant viruses and the parent virus, it is likely that the recombinant EV gene knockout viruses as well as the parent virus elicited cell-mediated immune responses that contributed to the protection of mice in this model.

Protection in this animal model was conferred by A33R/B5Rko in spite of impaired replication, as evidenced by reduced virus titers in cell culture but also in the reduced total vaccinia antibody response following vaccination, and the absence of an EV neutralizing antibody response in vivo. Regardless, it is still not clear whether these results can be extrapolated to conclude that an EV antibody response is not needed for an effective smallpox vaccine. As noted above, our experiments do not distinguish between an EV antibody response and a cell-mediated response to EV antigens. Further, there is likely a redundancy in the protective immune response, including both humoral and cellular immune responses, that could preclude the requirement for any individual vaccine antigens. Finally, there also remains the very real possibility that limitations inherent in the animal models used for preclinical vaccine evaluation do not faithfully predict the efficacy of candidate vaccines against smallpox in humans. Evaluation of the protective effect of the recombinant vaccine viruses generated in this study in other animal models of orthopoxvirus challenge may be informative. Nevertheless, in the absence of smallpox, and with good smallpox animal models not available nor practical, major challenges remain for the efficacy evaluation of new-generation smallpox vaccines that cannot be bridged to previous vaccines used successfully against smallpox.

## Supporting Information

Figure S1 **Characterization of Dryvax plaque isolates.**
Individual plaque clones of Dryvax were isolated, characterized, and compared to non-clonal Dryvax vaccine virus. (A) Viral DNA was isolated from BSC-1 cells infected with Dryvax and each of 6 Dryvax clones, digested with HindIII and analyzed by agarose gel electrophoresis. (B) BSC-1 cells were infected with Dryvax and Dryvax clones 3, 4, and 5 at a multiplicity of 0.01. Virus yield at 6, 24, and 48 hours was determined by plaque assay. (C) Groups of 5 mice were infected with 10^6^ pfu of Dryvax and Dryvax clones 3, 4, and 5, subcutaneously. Serum samples were obtained at 3 weeks after inoculation and total vaccinia-specific IgG determined by ELISA.
(TIF)
