# Supplementary figures and images for: Effect of the Deletion of Genes Encoding Proteins of the Extracellular Virion Form of Vaccinia Virus on Vaccine Immunogenicity and Protective Effectiveness in the Mouse Model
Source: PLoS One. 2013 Jun 13;8(6):e67984. doi: 10.1371/journal.pone.0067984 (PMC3681963; doi:10.1371/journal.pone.0067984)

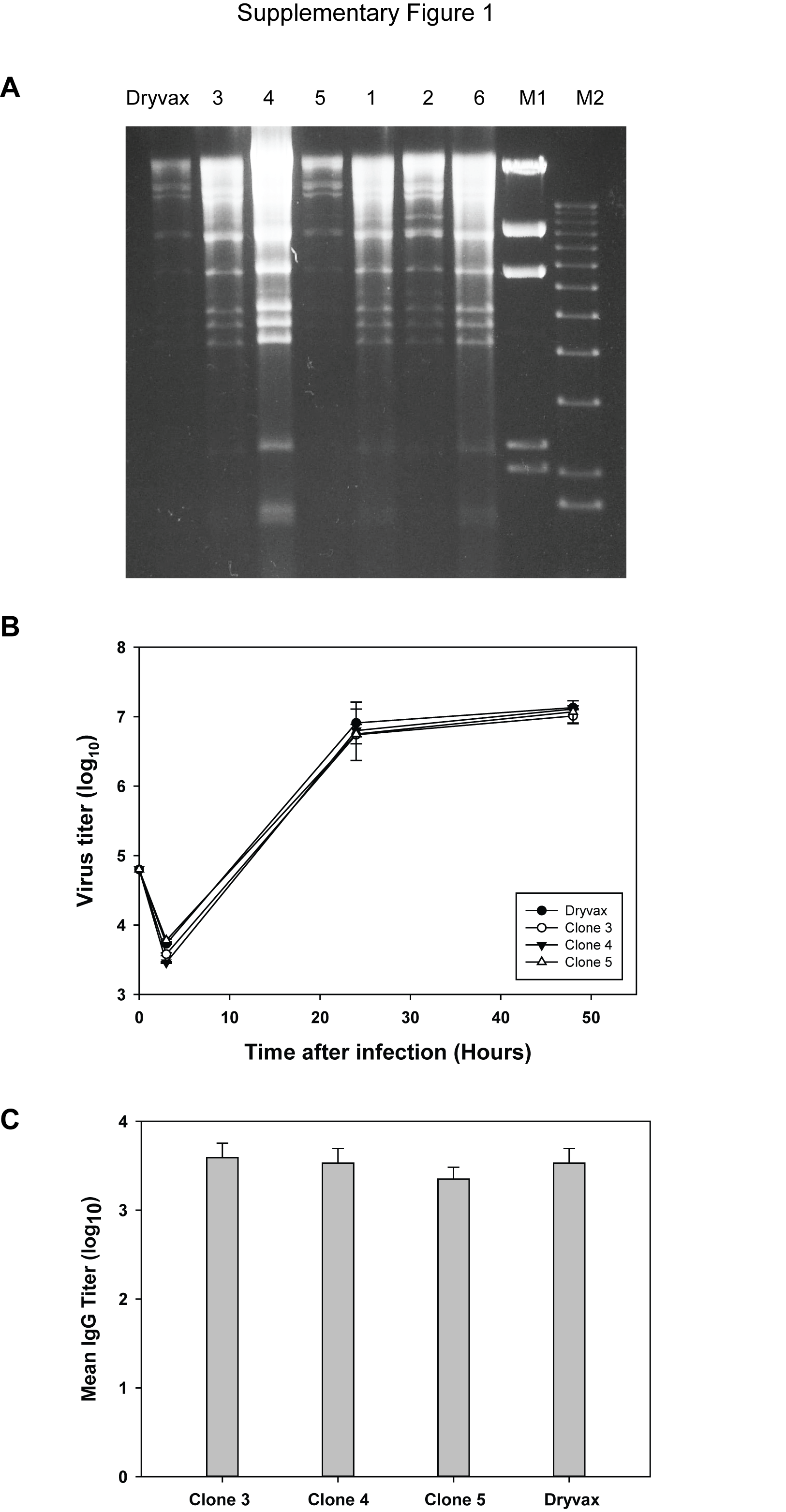

Supplement: Figure S1 — Characterization of Dryvax plaque isolates. Individual plaque clones of Dryvax were isolated, characterized, and compared to non-clonal Dryvax vaccine virus. (A) Viral DNA was isolated from BSC-1 cells infected with Dryvax and each of 6 Dryvax clones, digested with HindIII and analyzed by agarose gel electrophoresis. (B) BSC-1 cells were infected with Dryvax and Dryvax clones 3, 4, and 5 at a multiplicity of 0.01. Virus yield at 6, 24, and 48 hours was determined by plaque assay. (C) Groups of 5 mice were infected with 106 pfu of Dryvax and Dryvax clones 3, 4, and 5, subcutaneously. Serum samples were obtained at 3 weeks after inoculation and total vaccinia-specific IgG determined by ELISA. (TIF) [file pone.0067984.s001.tif]
